# Quantum dot imaging for embryonic stem cells

**DOI:** 10.1186/1472-6750-7-67

**Published:** 2007-10-09

**Authors:** Shuan Lin, Xiaoyan Xie, Manishkumar R Patel, Yao-Hung Yang, Zongjin Li, Feng Cao, Oliver Gheysens, Yan Zhang, Sanjiv S Gambhir, Jiang Hong Rao, Joseph C Wu

**Affiliations:** 1 Molecular Imaging Program at Stanford (MIPS) and Bio-X Program, Department of Radiology, Stanford University, Stanford, CA 94305, USA; 2 Department of Medicine, Division of Cardiology, Stanford University School of Medicine, Stanford, CA 94305, USA; 3 Department of Bioengineering, Stanford University, Stanford, CA 94305, USA

## Abstract

**Background:**

Semiconductor quantum dots (QDs) hold increasing potential for cellular imaging both *in vitro *and *in vivo*. In this report, we aimed to evaluate *in vivo *multiplex imaging of mouse embryonic stem (ES) cells labeled with Qtracker delivered quantum dots (QDs).

**Results:**

Murine embryonic stem (ES) cells were labeled with six different QDs using Qtracker. ES cell viability, proliferation, and differentiation were not adversely affected by QDs compared with non-labeled control cells (*P *= NS). Afterward, labeled ES cells were injected subcutaneously onto the backs of athymic nude mice. These labeled ES cells could be imaged with good contrast with one single excitation wavelength. With the same excitation wavelength, the signal intensity, defined as (total signal-background)/exposure time in millisecond was 11 ± 2 for cells labeled with QD 525, 12 ± 9 for QD 565, 176 ± 81 for QD 605, 176 ± 136 for QD 655, 167 ± 104 for QD 705, and 1,713 ± 482 for QD 800. Finally, we have shown that QD 800 offers greater fluorescent intensity than the other QDs tested.

**Conclusion:**

In summary, this is the first demonstration of *in vivo *multiplex imaging of mouse ES cells labeled QDs. Upon further improvements, QDs will have a greater potential for tracking stem cells within deep tissues. These results provide a promising tool for imaging stem cell therapy non-invasively *in vivo*.

## Background

**Figure 1:**
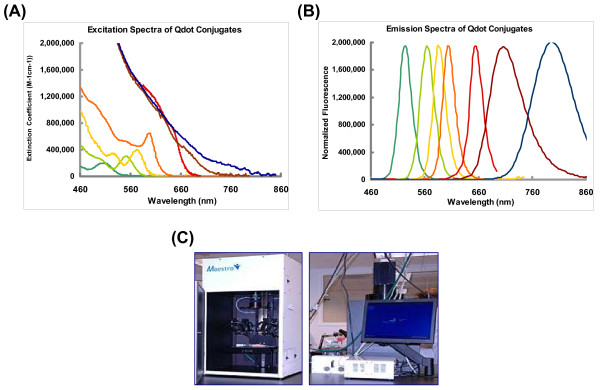
**Emission and excitation spectra of QDs (provided by Quamtum Corp.) and Maestro optical system**. (A) Excitation and (B) emission spectra of QDs used in the labeling experiments. Dark green = QD 525; green = QD 565; yellow = QD 585; orange = QD 605; red = QD 655; brown = QD 705; blue = QD 800. (C) The Maestro Optical imaging system.

Quantum dots (QDs) are emerging as an exciting new class of fluorescent probes for non-invasive *in vivo *imaging [1-5]. Compared to conventional organic dyes, QDs offer a number of fascinating optical and electronic properties. QDs are semiconductor nanocrystals that can be excited by a wide range of light, ranging from ultraviolet to near-infrared, and can emit different wavelengths of light, depending on their size and composition. QDs have broad excitation spectra and narrow emission spectra (Figure 1). Because QDs can be excited by one single wavelength and can emit light of different wavelengths, they are ideal probes for multiplex imaging [6]. By contrast, conventional organic dyes cannot be easily synthesized to emit different colors and have narrow excitation spectra and broad emission spectra that often cross into the red wavelengths, making it difficult to use these dyes for multiplexing. In addition, QDs have exceptional photostability (reviewed by Medintz *et al*. [7]). Due to their extreme brightness and resistance to photobleaching [8], QDs are ideal for live cell imaging wherein cells must be kept under the excitation light source for long periods of time. Their intense brightness is also helpful for single particle detection (reviewed by Michalet *et al*. [9]). By comparison, conventional dyes often photobleach, making longitudinal tracking difficult.

QDs' photophysical properties have broadened their application and shown great promise as imaging probes in bioimaging, drug discovery, and diagnosis. To keep up with their burgeoning utility, current QD technology has rapidly evolved. QDs have been used for tumor targeting and imaging [1], lymph node [3] and vascular mapping [5], and cellular trafficking [8,10]. QDs can be delivered in a targeted fashion by conjugating them with ligands and antibodies. QDs can also be introduced into cells non-specifically, which serves as a potential tracking marker for cellular imaging.

Stem cell therapy holds promise for treatment of intractable conditions such as Parkinson's disease, ischemic heart disease, diabetes, and degenerative joint diseases [11-14]. There are two types of cells used in stem cell therapy, adult stem cells and embryonic stem (ES) cells. Of the two, ES cells are the ultimate source for use in cell-based therapy because they posses a virtually unlimited capacity for self-renewal and pluripotency, which is defined as the ability to differentiate into all cell types, including neurons, cardiomyocytes, hepatocytes, islet cells, skeletal muscle cells, and endothelial cells [15]. In stem cell therapy, monitoring of cell survival and location after transplantation is important for determining their efficacy. Because the absorption and scattering of light in biological tissue can be considerable, any optical signal transmitted from deep tissues to the surface tends to diminish in strength (reviewed by Choy *et al*. [16]). With QDs' many advantages over traditional organic dyes, QDs may provide an excellent tool for imaging stem cell therapy.

In this study, we use the peptide-based reagent QTracker to label mouse ES cells with QDs and evaluate the utility of QDs for imaging stem cell therapy. We next show that labeling mouse ES cells with QDs does not adversely affect ES cell viability, proliferation, and differentiation. Finally, we examine QDs' potential for imaging ES cells *in vitro *and *in vivo*.

## Results

### Qtracker intracellular QD delivery

**Figure 2:**
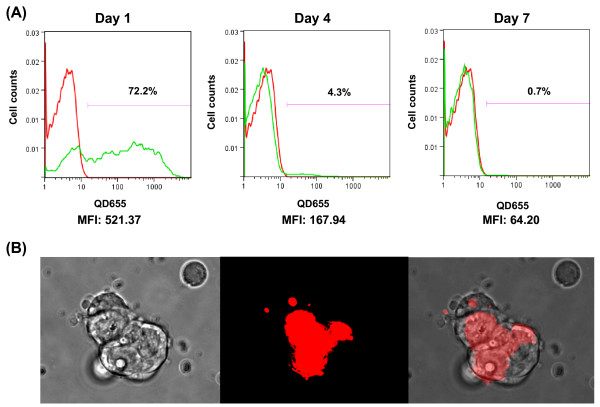
**Qtracker intracellular QD delivery quantified by flow cytometry**. (A) Flow cytometry detection of QD labeling of mouse ES cells on day 1, day 4, and day 7. Red line = unlabeled cells as control; green line = cells labeled with QD. (B) Fluorescent images of cells labeled with QDs on day 1 post labeling.

To deliver QDs, we used peptide-based QTracker, which has been shown to be an excellent and easy tool for study live cell mobility [17] and cell fusion [18]. In order to determine transfection efficiency in ES cells, labeled ES cells were analyzed by flow cytometry. Figure 2a shows a representative histogram plot based on forward scatter and side scatter gated cells. The red line shows fluorescence intensity of control unlabeled cells and the green line represents the labeled cells. As more QDs were taken up by these cells, the fluorescence intensity increased. Around 72% of the cells were positive 24 hours after labeling and the mean fluorescence intensity (MFI) was 521. However, by day 4 the percentage of positive cells had dropped to ~4% and by day 7 only ~0.7% of the cells were positive by FACS analysis when compared to control unlabeled cells. Fluorescence microscopy (Carl Zeiss Axiovert 200M) was used to image the cells on day 1. Representative brightfield and fluorescent images are shown in Figure 2b. ES cells can be labeled and monitored by FACS analysis up to 7 days.

### QDs do not affect ES cell viability and proliferation

**Figure 3:**
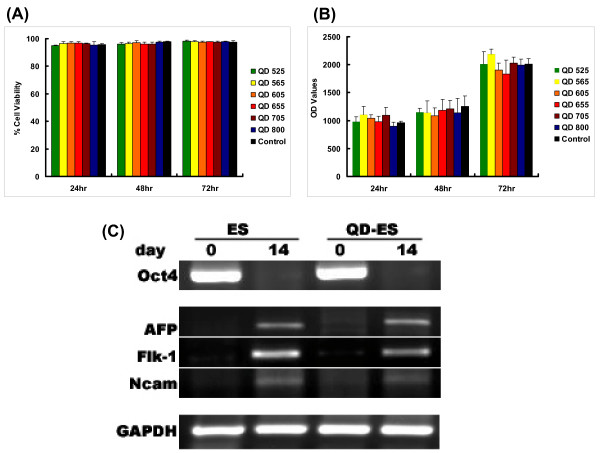
**Effects of QDs on ES cell viability, proliferation, and differentiation**. (A) Trypan blue exclusion assay and (B) CyQuant cell proliferation assay both showed no significant difference between unlabeled ES cells and labeled ES cell at 24, 48, and 72 hours. (C) RT-PCR analysis showed the levels of endoderm (AFP), mesoderm (Flk-1), and ectoderm (Ncam) germ layer marker increased from day 0 to day 14 of spontaneous ES cell differentiation using the hanging drop assay. The stem cell marker Oct4 decreased during the same period as expected. GAPDH is a loading control for all cells. Both QD labeled and unlabeled ES cells showed similar pattern on RT-PCR analysis.

Toxicity of QDs is a key factor in determining whether it will be a feasible probe for both cellular and clinical use. We carefully examined QDs' effect on ES cells by Trypan blue exclusion assay and a CyQuant proliferation assay. Figure 3a shows the percentage of live cells in triplicates at 24, 48 and 72 hours post QD labeling. Overall, there was no significant difference between labeled and unlabeled ES cells (*P *= NS) for all QDs that were tested: QD 525, 565, 605, 655, 705, and 800. To evaluate cell proliferation, we used the CyQuant assay, which measures the amount of nucleic acids in each well, thereby giving an accurate count of the number of cells in the experimental condition. As shown in Figure 3b, there was also no significant difference between QD labeled ES cells and unlabeled ES cells (*P *= NS).

### QDs have no profound effects on ES cell differentiation *in vitro*

Having demonstrated that QD labeling had no detectable effect on ES cell growth, we next tested its effect on cellular development and differentiation. Dubertret *et al*. showed that at high concentrations, QDs injected into an individual blastomere of Xenopus during very early cleavage stages can cause apparent abnormalities in late stage embryos [4]. Therefore, we examined the pluripotency of QD labeled mouse ES cells to ascertain if any developmental interference would occur. In the literature, both human and murine ES cells have well-documented differentiation and replication capacities [19,20]. Mouse ES cells were differentiated *in vitro *by hanging drop assay. We then isolated RNA samples from undifferentiated mouse ES cells and embryoid body at day 14 and analyzed them by RT-PCR. Both labeled and unlabeled undifferentiated ES cells (day 0) expressed ES cell specific marker Oct4. Likewise, both labeled and unlabeled differentiated ES cells (day 14) expressed specific markers for endoderm (alpha-1-fetoprotein, AFP), mesoderm (fetal liver kinase-1, Flk1), and ectoderm (neural cell adhesion molecule, Ncam) germ layers [21] (Figure 3c).

### *In vivo *multiplex imaging using QDs

**Figure 4:**
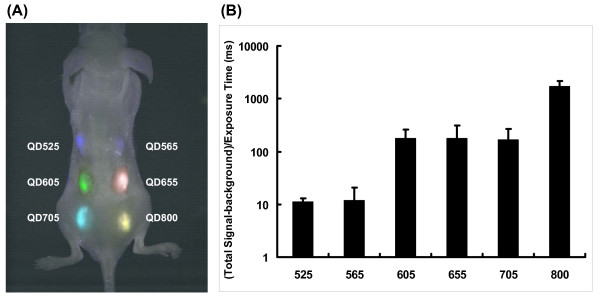
**Multiplex imaging capability of QD in live animals**. (A) 1 × 10^6 ^ES cells labeled with QD 525, 565, 605, 655, 705, and 800 were subcutaneously injected on the back of the athymic nude mice right after labeling and the image was taken with a single excitation light source right after injection. The quantification of fluorescent signal intensity defined as total signal-background/exposure time in millisecond was shown in (B).

One of the most attractive qualities of QDs is their capability for multiplex imaging (i.e., tracking different cell populations with different QDs using different emission wavelengths at the same time). In addition, as QDs are larger than organic dyes, they are not transferred between cells until the cells fuse. Therefore, QDs can provide an excellent tool for studying cell-cell interactions [18]. Here we used QD 525, 565, 605, 655, 705, and 800 to label 1 × 10^6 ^ES cells as described. Right after QD labeling, the labeled cells were subcutaneously injected into various locations on the back of athymic nude mice. Images were taken right after injection and the resulting stacked image shown in Figure 4a. The fluorescent intensity was directly proportional to the product of extinction coefficient and the quantum yield. Even though the QDs were excited by the same wavelength, the energy absorbed was different for each QD, causing some QDs to absorb less energy than others. This observation is due to the QDs' ability to produce different light levels at the same excitation wavelength as shown in Figure 1a. Therefore, QDs with longer emission wavelengths will appear brighter. With the same excitation wavelength, the signal intensity (defined as: (total signal-background)/exposure time in millisecond) was 11 ± 2 for cells labeled with QD 525, 12 ± 9 for QD 565, 176 ± 81 for QD 605, 176 ± 136 for QD 655, 167 ± 104 for QD 705, and 1,713 ± 482 for QD 800. Quantification of these results is shown in Figure 4b. In order to evaluate which QD was better for non-invasive imaging, we imaged the same transplanted mice longitudinally. After day 2, ES cells labeled with QD 525, 565, 605, 655, and 705 could not be detected *in vivo *using the Maestro system. In contrast, QD 800 signal could be detected up to 14 days in animals post injection, which is likely due to its higher extinction coefficient and wider emission spectra within near-infrared region.

### Detection sensitivity for *in vivo *imaging using QD800

**Figure 5:**
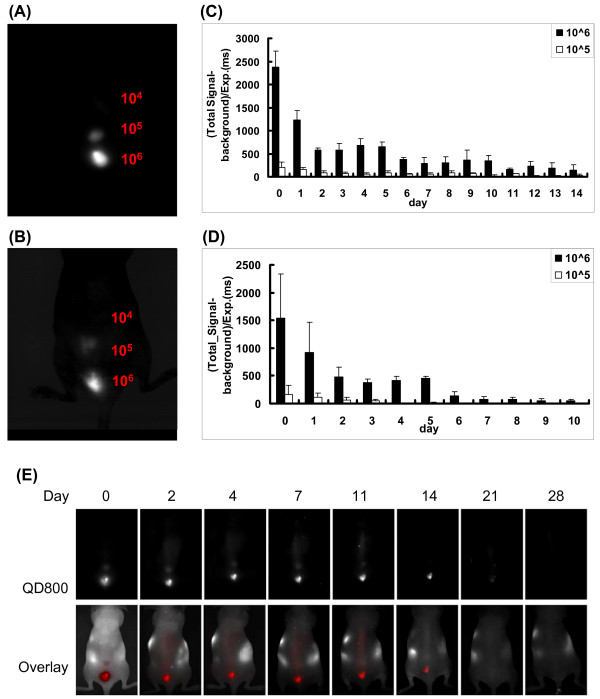
**Detection sensitivity of QD 800 imaging in live animals**. (A) 1 × 10^4^, 1 × 10^5 ^and 1 × 10^6 ^QD 800 labeled ES cells were subcutaneously injected on the back of the mice right after labeling. The image was taken 1 hour post injection with excitation filter 465 nm and emission filter 510 nm long-pass, and the quantification of the fluorescent intensity (total signal-background/exposure time (ms) was shown in (B). (C) After images were taken, the mice were imaged again with red excitation light source (640 nm) and the quantification of the fluorescent intensity was shown in (D). Longitudinal imaging of the same representative animal for 1 month shows detection of QD signals up to day 14 (E).

We have shown that QD 800 offers greater fluorescent intensity than the other QDs tested. However, its detection sensitivity is currently unknown. In particular, what are the fewest number of labeled cells that can be detected by the Maestro system and for what duration? In order to determine the detection sensitivity for *in vivo *imaging, we subcutaneously injected different numbers of QD 800 labeled ES cells (1 × 10^4^, 1 × 10^5^, and 1 × 10^6^) into the back of the mice right after labeling. Images were taken 1 hour post injection and then daily thereafter for 2 weeks using the Maestro Optical imaging system (excitation: 465 nm, emission: 515 long-pass). Figure 5a showed that ~1 × 10^5 ^subcutaneously injected QD labeled cells could be seen through the Maestro system. The signal intensity quantification is shown in Figure 5b. Since QD 800 could also be excited by red light, which offered better tissue penetration, we also imaged the mice using excitation filter 640 nm and emission filter 700 long-pass (Figure 5c). We compared the resulting image to that obtained from earlier settings. Although we still could not visualize the 1 × 10^4 ^labeled cells, the signal intensity from 1 × 10^6 ^labeled cells did increase with the red light excitation (from 1538 ± 793 to 2378 ± 352) (Figure 5d). Again, signals were still present in the animals up to day 14 using excitation filter 640 nm as shown in Figure 5e.

### Postmortem histologic analysis of QD labeled ES cells

**Figure 6:**
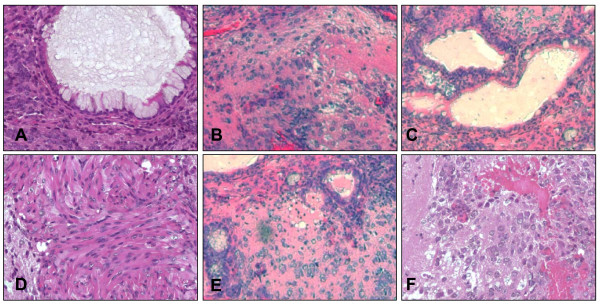
**Postmortem histological analysis of transplanted ES cells**. (A,D) respiratory epithelium with ciliated columnar and mucin producing goblet cells; (B,E) osteochondroid formation; (C) squamous cell differentiation with keratin pearl; and (F) immature brain-like neural cell formation.

After imaging, animals were sacrificed and the subcutaneous tumor developed from 1 × 10^6 ^QD800 labeled ES cells was removed for detailed postmortem analysis at day 28 post-injection. Conventional histology using H&E stains confirmed the intact *in vivo *differentiation ability of QD labeled ES cells in living animals (Figure 6). These *in vivo *histologic data are concordant with previous *in vitro *RT-PCR data shown in Figure 4, which further suggest that QDs do not affect the developmental pluripotency of ES cells. However, we could not observe any QDs under microscopic level at day 28, likely due to dilution and diffusion effects.

## Discussion

Stem cells offer an exciting new branch of therapy to treat a variety of conditions and diseases. It is therefore important to develop methods to monitor cell survival and location after transplantation. Due to its many advantages over conventional organic dyes, QDs serve as good candidates to monitor these parameters. In order to evaluate their *in vivo *ability, we delivered them by using commercially available QTracker. Strategies for *ex vivo *cell labeling by QDs include non-specific endocytosis, microinjection, liposome mediated uptake, electroporation, and peptide-based reagents. Previous studies have shown that the liposome-based reagent Lipofectamin 2000 had the highest delivery efficiency, but the QDs were delivered in aggregates [22]. Electroporation also delivered QDs in aggregates [22], and may even cause cell death. Peptide-based QTracker [23] reagents (Invitrogen, CA) deliver QDs into the live cells, and have been shown to be an excellent and easy tool for studying live cell mobility [17] and cell fusion [18].

In this report, we evaluated ES cells labeled with QDs using commercially available Qtracker for non invasive *in vivo *imaging in living mice. Twenty-four hours after labeling ES cells with QDs, 72% of the cells were positive. However, by day 4 the percentage of positive cells dropped to 4%. This dramatic decrease could be due to the rapid division of ES cells (doubling time of 12 – 15 hours) or QD diffusion out of dividing cells over time thus causing a dilution of QD signal. The dramatic decrease in signal is consistent with a previous study that used QDs to label human cervical adenocarcinoma cells [10].

Another important question is whether QDs affect ES cell properties (i.e., pluripotency and self-renewal) that make them an attractive choice for regenerative therapy. Previous studies have shown that QD toxicity is dose dependent with increasing concentrations affecting cell growth and viability [24]. However, we were interested in any toxicity caused at concentrations used for labeling cells for *in vivo *applications. Therefore, we examined ES cell proliferation and viability at one QD concentration (10 nM) and observed no significant changes between QD labeled ES cells and control unlabeled ES cells. This was true for all QDs tested: QD 525, 565, 605, 655, 705, and 800. These results concur with the study by Jaiswal *et al*. that also showed no adverse effects by QDs on the viability, morphology, function, and development of various other cells [10]. Likewise, we confirmed that QDs also had no adverse affect on ES cell differentiation based on RT-PCR analysis of germ layer specific genes. Implanted ES cells are known to form teratoma tumors with a variety of differentiated tissues [25]. In Figure 6, we found that the teratoma consisted of a variety of tissues including respiratory epithelium, osteochondroid, squamous cell, and immature brain-like neural cell based on histology. This confirmed that QD labeling did not affect *in vivo *differentiation as well. However, although ES cell-derived teratomas were retrieved from the animals, they were not shown to be QD labelled. We believe that the *in vivo *signal could be due to uptake of QD by neighboring host cells. Thus, the poor retention of QDs in targets cells may be a problem for long-term tracking, and more detailed analysis are needed to address this issue in the future.

Another advantage of QDs is their ability to do multiplex imaging of different QDs at the same time. However, in our study, ES cells labeled with different QDs were only capable of being imaged up to day 2 after subcutaneous implantation. A likely cause for this could be the loss of signal due to rapid cell division. Another possible cause could be serum instability of the QDs. Cai *et al*. reported that QD 705 lost 14% of its original intensity after 24 hours of incubation in mouse serum [26]. Any loss of signal could hamper detection of QD labeled cells at later time points, especially those that are not within the near-infrared region since signals from these QDs will also be mostly absorbed by the skin. For those QDs that are in near-infrared region, QD 705 and QD 800, the difference in intensity could be due to transfection efficiency since these two QDs have similar extinction coefficients and quantum yield according to the manufacturer. However, extinction coefficients and quantum yield data were obtained *in vitro *and not in an animal setting. Moreover, the transfection efficiency was similar across all QDs. Therefore, we believe transfection efficiency is unlikely to be the cause of the difference in intensity observed *in vivo*. Due to its higher extinction coefficient and wider emission spectra within near-infrared region, only QD 800 signals were capable to be imaged in the animals for up to 14 days. We observed an increase in signal intensity when using a red shifted excitation laser (640 nm) to image QD 800 labeled ES cells. The normal excitation wavelength is 465 nm. This was somewhat surprising since the excitation coefficient of QD 800 is lower at 640 nm than it is at 465 nm. That is at 640 nm, QD 800 absorbs light with less efficiency than at 465 nm, so less QDs become excited and thus give off lower signal intensities. However, the tissue penetration is much greater at 640 nm. Therefore, labeled cells that would not have been excited at 465 nm could be excited at 640 nm. Thus, these newly excited cells could contribute to the greater signal intensity seen at the detection wavelength of 800 nm.

## Conclusion

In summary, we report the successful demonstration of labeling ES cells with QDs and imaging these labeled cells *in vivo*. We have shown that it is feasible to label ES cells with QDs by Q-Tracker with high efficiency. After labeling, QDs did not affect the viability, and proliferation of ES cells, and have no profound effect on differentiation capacity of ES cells within the sensitivities of the screening assays used. We tested multiplex imaging *in vivo *using the Maestro system and showed that QD 525, QD 565, QD605, QD 655, QD 705, and QD 800 labeled ES cells can be detected *in vivo *using a single excitation wavelength (465 nm). This versatility makes them good candidates for tumor targeting [1], lymph node [3] and vascular mapping [5], and cell trafficking [8,10] in small animal imaging. Nevertheless, the use of QD in stem cells is only beginning to be explored. To our knowledge, this is the first demonstration of *in vivo *multiplex imaging of mouse ES cells labeled QDs. Upon further improvements (e.g., near-infrared QDs, better serum stability, and improved cell retention), QDs will have greater potential for tracking of stem cells within deep tissues.

## Methods

### Culture of undifferentiated ES cells

The murine ES-D3 cell line (CRL-1934) was obtained from the American Type Culture Collection (ATCC; Manassas, VA). ES cells were kept in an undifferentiated, pluripotent state with 1000 IU/ml leukemia inhibitory factor (LIF; Chemicon. ESGRO, ESG1107) and grown on top of the murine embryonic fibroblasts feeder layer inactivated by 10 ug/ml mitomycin C (Sigma). ES cells were cultured on 0.1% gelatin-coated plastic dishes in ES medium containing Dulbecco Modified Eagle Medium supplemented with 15% fetal calf serum, 0.1 mmol/l β-mercaptoethanol, 2 mmol/l glutamine, and 0.1 mmol/l nonessential amino acids as described previously [27,28].

### Flow cytometry and fluorescent microscopy

Trypsinized mouse ES cells were labeled with QD 655 (10 nM) using Qtracker according to the manufacturer's protocol. Briefly, 10 nM of labeling solution was prepared according to the kit direction. Trypsinized mouse ES cells (1 × 10^6^) were added to the 0.2 ml of labeling solution. After incubating at 37°C for 60 minutes with intermittent mixing, the ES cells were washed twice with PBS to remove any free QDs and plated on 0.01% gelatin coated plates. Fluorescence microscopy (Carl Zeiss Axiovert 200M) was used to image the cells on day 1. Labeled ES cells were analyzed by flow cytometry (FACSCalibur; BD Biosciences, San Jose, CA) using the FL3 channel to detect QD 655 labelled cells on days 1, 4, and 7 post-labeling. Acquisition data were analyzed by the FlowJo software.

### Effect of QDs on ES cell viability and proliferation

ES cells labeled with six different QDs (10 nM each) and control unlabeled ES cells were plated uniformly in 96-well plates at a density of 5,000 cells per well. Cells were treated according to the manufacturer's protocol and read out on a fluorescence microplate reader (SpectraMax Gemini EM, Molecular Devices Corporation, Sunnyvale, CA) at 24, 48, and 72 hours post labeling. For Trypan blue exclusion assay (indicative of cell death), aliquots of labeled cells were removed at specific time points and mixed with Trypan blue. The number of dead cells was determined by counting blue cells under a light microscope.

### Embryoid body formation and differentiation

ES cells were differentiated *in vitro *by the "hanging drop" method as described previously [28-30]. Briefly, the main steps included withdrawal of LIF and cultivation of 400 cells in 18 μl hanging drops to produce embryoid bodies for 3 days, followed by cultivation as suspension in ultra-low-cluster 96-well flat-bottom plates for 2 days. Next, the embryoid bodies were seeded onto 48-well plates.

### RT-PCR analysis of embryonic and germ layer specific transcripts

Reverse-transcription polymerase chain reaction (RT-PCR) was used to compare the expression of embryonic marker (Oct4), endoderm (alpha-1-fetoprotein, AFP), mesoderm (fetal liver kinase-1, Flk1), and ectoderm (neural cell adhesion molecule, Ncam) germ layer markers [21] between control unlabeled ES and QD-labeled ES cells. Total RNA was prepared from cells with Trizol reagent (Invitrogen) according to the manufacturer's protocol. The primer sets used in the amplification reaction were as follow: Oct4 forward primer GGCGTTCTCTTTGCAAAGGTGTTC, reverse primer CTCGAACCACATCCTTCTCT; AFP forward primer TATCAGCCACTGCTGCAACT, reverse primer GTTCAGGCTTTTGCTTCACC; Flk1 forward primer CACCTGGCACTCTCCACCTTC, reverse primer GATTTCATCCCACTACCGAAAG; Ncam forward primer GGAAGGGAACCAAGTGAACA, reverse primer ACGGTGTGTCTGCTTGAACA. PCR products were separated on 1% agarose gel electrophoresis and quantified with Labworks 4.6 Image Acquisition and analysis software (UVP Bio-Imaging Systems).

### *In vivo *fluorescence imaging of QD-labeled ES cells

Right after labeling ES cells with QDs by QTracker, the labeled cells were subcutaneously injected with Matrigel (50 μl, vol. 1:1, BD Biosciences, San Jose, CA) into various locations on the back of athymic nude mice (n = 6). Images were taken with an excitation filter of 465 nm and an emission filter of 510 nm long-pass using the Maestro Optical imaging (CRI Inc, Woburn, MA) as shown in Figure 1b. Detection was set to capture images automatically at 10 nm increments from 500 to 850 nm. The vendor's software (Nuance 2p12_beta) determined the correct exposure time for each QD labeled cells. The resulting TIFF image was loaded into the software and analyzed. Spectral unmixing was done using a user-defined library according to manufacturer's direction for each QD. Briefly, images of six different QD labeled ES cells in 1.5 ml micro-centrifuge tubes were taken separately. Each QD library spectra was decided and set by unmixing autofluorescence spectra and QD spectra manually selected from the image using the computer mouse to select appropriate regions. Images for QD800 sensitivity experiment was taken with an excitation filter of 640 nm and an emission filter of 700 nm long-pass.

### Postmortem immunohistochemical stainings

After imaging, all animals were euthanized by protocol approved by the Stanford Animal Research Committee. Explanted subcutaneous teratomas were routinely processed for hematoxylin-and-eosin staining. Slides were interpreted by an expert pathologist blinded to the study (AJC).

### Statistical analysis

Data were presented as mean ± SD. For Statistical analysis, the 2-tailed Student *t *test was used. Differences were considered significant at *P *< 0.05.

## Authors' contributions

SL carried out the QTracker transfection efficiency studies (FACS and fluorescence imaging), the cell viability assay, the ES cell differentiation assay, the *in vivo *multiplex imaging, and the *in vivo *sensitivity study, participated in the cell proliferation assay, and drafted the manuscript. XX carried out the cell proliferation studies, the histology studies, and RT-PCR analysis, and participated in the *in vivo *multiplex imaging and the *in vivo *sensitivity studies. MRP participated in the *in vivo *multiplex imaging, and the *in vivo *sensitivity study, and helped to draft the manuscript. YY and ZL participated in the QTracker transfection efficiency studies. FC and OG participated in the *in vivo *multiplex imaging and *in vivo *sensitivity study. YZ participated in the QTracker transfection efficiency studies. SSG and JHR participated in the design of the study and helped to draft the manuscript. JCW conceived the study, and participated in its design and coordination and helped to draft the manuscript. All authors have read and approved the final manuscript.
